# Overadjustment in Regression Analyses: Considerations When Evaluating Relationships Between Body Mass Index, Muscle Strength, and Body Size

**DOI:** 10.1093/gerona/glt186

**Published:** 2013-12-03

**Authors:** Victoria L. Keevil, Kay-Tee Khaw

**Affiliations:** Department of Public Health and Primary Care, University of Cambridge, UK.

Dear Editor,

We are writing in response to the article published in your journal by Stenholm and colleagues entitled ‘Association between obesity history and hand grip strength in older adults—exploring the roles of inflammation and insulin resistance as mediating factors’. *J Gerontol A Biol Sci Med Sci.* 2011;66A(3):341–348.

This article explores the association between obesity, defined as body mass index (BMI) ≥30 kg/m^2^, and hand grip strength. The authors report that obese men and women (*n* = 2,021) had lower grip strength than nonobese participants. Additionally, using recall of past weight to retrospectively calculate BMI across adulthood, a dose–response association was observed between longer obesity duration and lower grip strength.

These results conflict with those of other groups who have not reported inverse associations between BMI and grip strength (1,2). However, all linear regression analyses in Stenholm’s article were adjusted for body weight, to account for differences in muscle strength associated with increasing body size. Logistic regression models were also adjusted for body weight by virtue of the relative outcome measure used (grip strength/weight). The linear regression equation describing the simplest model reported in the article (Table 2, model 1: never obese vs currently obese) can be written as:


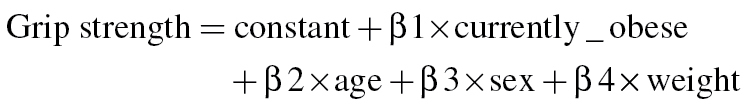


Given that obesity is defined using BMI, this could also be written as:


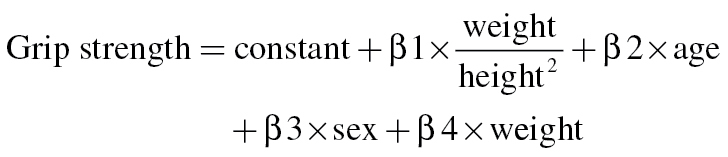


When written out in this way, it is clear that overadjustment in analyses is a concern. The coefficient β1 represents the difference in grip strength between participants with a BMI ≥ 30 kg/m^2^ and those with a BMI < 30 kg/m^2^, when age, sex, and weight are held constant. However, if weight is held constant, BMI can only differ if height varies between the two groups considered. Thus, increasing BMI is simply an indicator of lower height, rather than obesity, and the inverse association reported mainly indicates a positive association of height with grip strength.

We can illustrate this using data from the third health examination (3HC) of our own cohort study, the European Prospective Investigation into Cancer (EPIC)-Norfolk (3). Table 1 shows the mean maximum grip strength and height of men and women by tertile of BMI. When all participants are considered together, grip strength increases with increasing BMI and height does not differ between categories. However, when participants are stratified into tertiles of weight, grip strength decreases with increasing BMI and increasing BMI is now also associated with decreasing height.

**Table 1.** Mean Maximum Grip Strength (kg) of Men and Women From the 3HC of EPIC-Norfolk Across BMI Categories, After Stratification of Participants into Sex-Specific Tertiles of Weight and Adjustment for Age

Weight Tertiles	*N*	BMI Tertiles					
		1		2		3	
		Mean maximum grip strength, in kg (*SE*)	Height, in cm	Mean maximum grip strength, in kg (*SE*)	Height, in cm	Mean maximum grip strength, in kg (*SE*)	Height, in cm
Men							
All	3,797	38.0 (0.20)	174	39.3 (0.20)	173	40.0 (0.20)	173
1	1,259	37.4 (0.23)	171	36.9 (0.40)	166	35.4 (1.32)	159
2	1,272	39.7 (0.39)	180	39.5 (0.26)	173	38.5 (0.42)	168
3	1,266	42.3 (1.40)	190	41.4 (0.41)	182	40.6 (0.22)	175
Women							
All	4,644	23.9 (0.13)	161	24.4 (0.13)	161	24.7 (0.13)	160
1	1,537	23.6 (0.14)	160	22.7 (0.28)	153	20.2 (1.20)	147
2	1,547	25.0 (0.28)	167	24.5 (0.16)	161	23.6 (0.30)	154
3	1,560	26.5 (1.56)	177	25.8 (0.30)	168	25.0 (0.14)	161

*Notes:* Weight tertiles—men: ≤75.6, 75.7–85.4, and ≥85.5 kg; women: ≤62.0, 62.1–71.7, and ≥71.8 kg. BMI tertiles—men: <25.4, 25.4–28.1, and >28.1kg/m^2^; women: <24.2, 24.2–27.7, and >27.7 kg/m^2^. BMI = body mass index.

Furthermore, after characterizing participants as obese and nonobese using the same BMI cut point as Stenholm, we found that the direction and strength of association between “obesity” and grip strength changed with adjustment for weight. In cross-sectional analyses, obese participants were 0.71 kg (95% confidence interval 0.38, 1.04) *stronger* than nonobese participants after adjustment for age and sex but were 2.31 kg (95% confidence interval 1.87, 2.75) *weaker* after further adjustment for weight.

In summary, it is important to consider the consequences of adjusting for covariates in statistical models. In this example, it was reasonable to consider the influence of body size variations unrelated to fat accumulation on the association being investigated, but the method employed was flawed. When using BMI as the adiposity measure, it is inappropriate to adjust analyses for weight, since this creates an inverse association between BMI and height and renders regression models difficult to interpret. This leads to uncertainty that the data presented by Stenholm and colleagues can support the conclusions made, even though the conclusions themselves may be valid.

## Funding

The EPIC-Norfolk study is funded by program grants from the Medical Research Council (G0401527) and Cancer Research UK (C864/A8257) while the 3HC clinic was funded by a grant from Research into Ageing (262). V.L.K. is funded by a Wellcome Trust Clinical Training Fellowship (092077/Z/10/Z).

## References

[1] Rolland Y Lauwers-Cances V Pahor M Fillaux J Grandjean H Vellas B Muscle strength in obese elderly women: effect of recreational physical activity in a cross-sectional study. Am J Clin Nutr. 2004;79(4):552–557 15051596 10.1093/ajcn/79.4.552

[2] Hardy R Cooper R Aihie Sayer A Body mass index, muscle strength and physical performance in older adults from eight cohort studies: the HALCyon programme. PLoS One. 2013;8(2):e56483. 10.1371/journal.pone.0056483 23437142 10.1371/journal.pone.0056483 PMC3577921

[3] Hayat SA Luben R Keevil VL Cohort profile: a prospective cohort study of objective physical and cognitive capability and visual health in an ageing population of men and women in Norfolk (EPIC-Norfolk 3). Int J Epidemiol. 2013:1–10. 10.1093/ije/dyt086 23771720 10.1093/ije/dyt086 PMC4121549

